# The Draft Genome of the Non-Host-Associated *Methanobrevibacter arboriphilus* Strain DH1 Encodes a Large Repertoire of Adhesin-Like Proteins

**DOI:** 10.1155/2017/4097425

**Published:** 2017-05-28

**Authors:** Anja Poehlein, Rolf Daniel, Henning Seedorf

**Affiliations:** ^1^Genomic and Applied Microbiology & Göttingen Genomics Laboratory, Georg-August University, Göttingen, Germany; ^2^Temasek Life Sciences Laboratory, Singapore; ^3^Department of Biological Sciences, National University of Singapore, Singapore 117604

## Abstract

*Methanobrevibacter arboriphilus* strain DH1 is an autotrophic methanogen that was isolated from the wetwood of methane-emitting trees. This species has been of considerable interest for its unusual oxygen tolerance and has been studied as a model organism for more than four decades. Strain DH1 is closely related to other host-associated *Methanobrevibacter* species from intestinal tracts of animals and the rumen, making this strain an interesting candidate for comparative analysis to identify factors important for colonizing intestinal environments. Here, the genome sequence of *M. arboriphilus* strain DH1 is reported. The draft genome is composed of 2.445.031 bp with an average GC content of 25.44% and predicted to harbour 1964 protein-encoding genes. Among the predicted genes, there are also more than 50 putative genes for the so-called adhesin-like proteins (ALPs). The presence of ALP-encoding genes in the genome of this non-host-associated methanogen strongly suggests that target surfaces for ALPs other than host tissues also need to be considered as potential interaction partners. The high abundance of ALPs may also indicate that these types of proteins are more characteristic for specific phylogenetic groups of methanogens rather than being indicative for a particular environment the methanogens thrives in.

## 1. Introduction

Methanogenic archaea (methanogens) comprise a phylogenetically diverse group of microorganisms that can grow in a wide variety of different anoxic environments, such as sediments or the intestinal tracts of animals and humans. The energy metabolism of the two different types of methanogens, those that contain and those that lack cytochromes, has been investigated in detail [1]; however, it remains poorly understood how methanogens respond to some specific environmental stimuli or how they physically interact with their environment. The latter is of particular interest for members of the genus *Methanobrevibacter* of the order Methanobacteriales. Most *Methanobrevibacter* species grow by using the same highly conserved hydrogenotrophic methanogenesis pathway, with the genes for this pathway conserved across different *Methanobrevibacter* species [1–3]. Yet some species of this genus appear to be more indicative for certain environments than others. For example, previous studies have shown that the methanogen communities in the rumens of cattle and sheep are dominated by two different *Methanobrevibacter* species, *M. gottschalkii* and *M. ruminantium* [4, 5], while microbiota analyses of the human gut revealed that *M. smithii* is the predominant methanogen in this habitat [6–8]. The growing number of sequenced *Methanobrevibacter* genomes allows performing comparative genome analysis in order to identify traits in strains and species that have adapted to a specific environment. It must be noted, however, that all of these genomes have been derived from methanogens that are associated either with the human or animal intestinal tract.

One exception is *M. arboriphilus* strain DH1 [9], initially named *Methanobacterium arbophilicum* [10], as this strain does not live in association with an animal or human host. This differentiating characteristic turns *M. arboriphilus* strain DH1 into ideal candidate for comparative analyses between genomes of host-associated and non-host-associated *Methanobrevibacter* species. Type strain DH1 was cultivated in 1975 from wetwood of living trees and was considered to be the source of methane emissions from these trees [10]. Since its first isolation, other strains of *M. arboriphilus* have been isolated from a variety of different environments, such as digested sewage sludge [11], rice paddy soil [12], and more recently the human gut [13]. The gene repertoire of the different *M. arboriphilus* strains and their adaptations to specific environments as well as the similarity of these different *M. arboriphilus* strains to each other remains largely unknown.

One remarkable physiological feature of *M. arboriphilus* strain DH1 is its unusually high oxygen tolerance. This was unexpected, as methanogens and especially most mesophilic members of the cytochrome-lacking methanogen orders were predicted to be particularly oxygen-sensitive due to the lack of cytochrome-oxidase and catalase, two heme proteins. However, physiological characterization of *M. cuticularis* and *M. curvatus* from termites and of different *M. arboriphilus* strains revealed catalase activity in these organisms [14, 15]. In subsequent experiments, it was shown that *M. arboriphilus* produced a functional heme catalase when the medium was supplemented with hemin [15]. The absence of heme biosynthesis in *Methanobrevibacter* species and the analysis of the *M. arboriphilus* catalase gene suggested an acquisition of this gene via horizontal gene transfer [15]. The presence of the catalase gene in some *Methanobrevibacter* and its absence in others (e.g., *M. smithii* and *M. ruminantium*) represent just one example of differential adaptation to a specific environmental condition within the genus.

How *Methanobrevibacter* species have adapted to specific hosts is not well understood. The analysis of the first genome sequence of a host-associated methanogen, *Methanosphaera stadtmanae*, revealed some intriguing features in this regard [16]. It was found that the genome of this methanogen encoded almost 40 predicted proteins that were not encoded at all or only in smaller numbers by the genomes of non-host-associated methanogens. These proteins, designated *Asn-Thr-rich proteins*, are predicted to be longer than the mean protein length, anchored in the membrane via a C- or N-terminal helix and oriented towards the extracellular space, and have an eponymous overrepresentation of asparagine and threonine in their amino acid composition [16]. Meanwhile, several genomes of host-associated *Methanobrevibacter* strains have been sequenced and their analysis revealed an ubiquitous presence and high abundance of genes encoding *Asn-Thr-rich proteins* [13, 17–22]. This and the remote similarity of these proteins to bacterial homologues led to the annotation of these proteins as adhesin-like proteins (ALPs) [22]. The role for most ALPs remains speculative as there is only limited functional evidence that these proteins act as adhesins and what their targets could be [8]. However, their high abundance in host-associated methanogens suggests a potential role in methanogen-vertebrate host interaction. This hypothesis would be corroborated if ALPs were present only in small numbers or completely absent from non-host-associated *Methanobrevibacter* strains, such as *M. arboriphilus* strain DH1.

Here, the genome sequence of *Methanobrevibacter arboriphilus* strain DH1 is described and compared to the genomes of seven other *Methanobrevibacter* species (including *M. arboriphilus* strain ANOR1 (isolated from human feces [13])) and *Methanosphaera stadtmanae*. The genome analysis and comparisons revealed that *M. arboriphilus* strain DH1 not only has maintained all genes essential for autotrophic growth but it also shares some genomic features with closely related host-associated methanogens.

## 2. Materials and Methods

### 2.1. Cultivation of Microorganism and DNA Extraction

*Methanobrevibacter arboriphilus* strain DH1 (DSM 1125) was obtained from the Deutsche Sammlung von Mikroorganismen und Zellkulturen (DSMZ), Braunschweig, Germany. The methanogenic archaeon was cultivated under anaerobic conditions using a medium described by Asakawa et al. [12]. DNA was isolated from the enrichment culture by phenol-chloroform extraction followed by a QIAquick column (Qiagen, Hilden, Germany) purification [23, 24].

### 2.2. Genome Sequencing

The genome of *Methanobrevibacter arboriphilus strain DH1* was sequenced with a combined approach using the 454 GS-FLX Titanium XL system (titanium GS70 chemistry, Roche Life Science, Mannheim, Germany) and the GenomeAnalyser IIx (Illumina, San Diego, CA). Shotgun libraries were prepared according to the manufacturer's protocols, resulting in 99,511 454 shotgun sequencing reads and 11,827,196 112 bp paired-end Illumina sequencing reads. The Illumina reads were quality trimmed using Trimmomatic version 0.32 [25]. All of the 454 shotgun reads and 2,637,606 of the Illumina reads were used for the initial hybrid de novo assembly with MIRA 3.4 [26] and Newbler 2.8 (Roche Life Science, Mannheim, Germany). The final assembly contained 40 contigs with an average coverage of 92.85. The assembly was validated and the read coverage determined with QualiMap version 2.1 [27]. The quality and the completeness of the draft genome has been validated with CheckM [28].

### 2.3. Sequence Annotation

The genome data were uploaded to the Integrated Microbial Genomes Expert Review (IMG/ER) platform (https://img.jgi.doe.gov/cgi-bin/er/main.cgi). Coding sequences were predicted and annotated using the automated pipeline of IMG/ER [29]. Briefly, protein-encoding genes were identified with GeneMark [30], and candidate homologue genes of the genomes were computed using BLASTp [31]. Automated annotations of coding sequences were verified and curated by comparing various annotations based on functional resources, such as COG clusters, Pfam, TIGRfam, and gene ontology. The annotated genome sequence of *M. arboriphilus* strain DH1 (Gs0106968 or Gp0076455) is available in the Genomes Online database (http://www.genomesonline.org/).

### 2.4. Nucleotide Sequence Accession Number

The annotated genome of *Methanobrevibacter arboriphilus strain DH1* has been deposited at DDBJ/EMBL/GenBank under the accession JXMW00000000. The version described in this paper is version JXMW01000000.

### 2.5. Tree Construction and Taxonomic Assignment

Aligned sequences were selected from RIM-DB [5] and exported in phylip format to construct phylogenetic trees using all available base positions. Maximum likelihood phylogenetic trees based on aligned archaeal 16S rRNA gene sequences were generated using RAxML version 7.0.3 [32]. The parameters “-m GTRGAMMA -# 500 -f a -x 2 -p 2” were used.

### 2.6. Genome Comparison

Orthologous genes (orthologs) among genome sequences were identified using Proteinortho version 4.26 (default specification: BLAST = BLASTp v2.2.24, *E* value = 1*e*^−10^, alg.-conn. = 0.1, coverage = 0.5, percent_identity = 50, adaptive_similarity = 0.95, inc_pairs = 1, inc_singles = 1, selfblast = 1, and unambiguous = 0) [33]. COG categories of the genes were extracted from IMG database entries of *M. arboriphilus* DH1.

## 3. Results and Discussion

### 3.1. General Features

**Figure 1:**
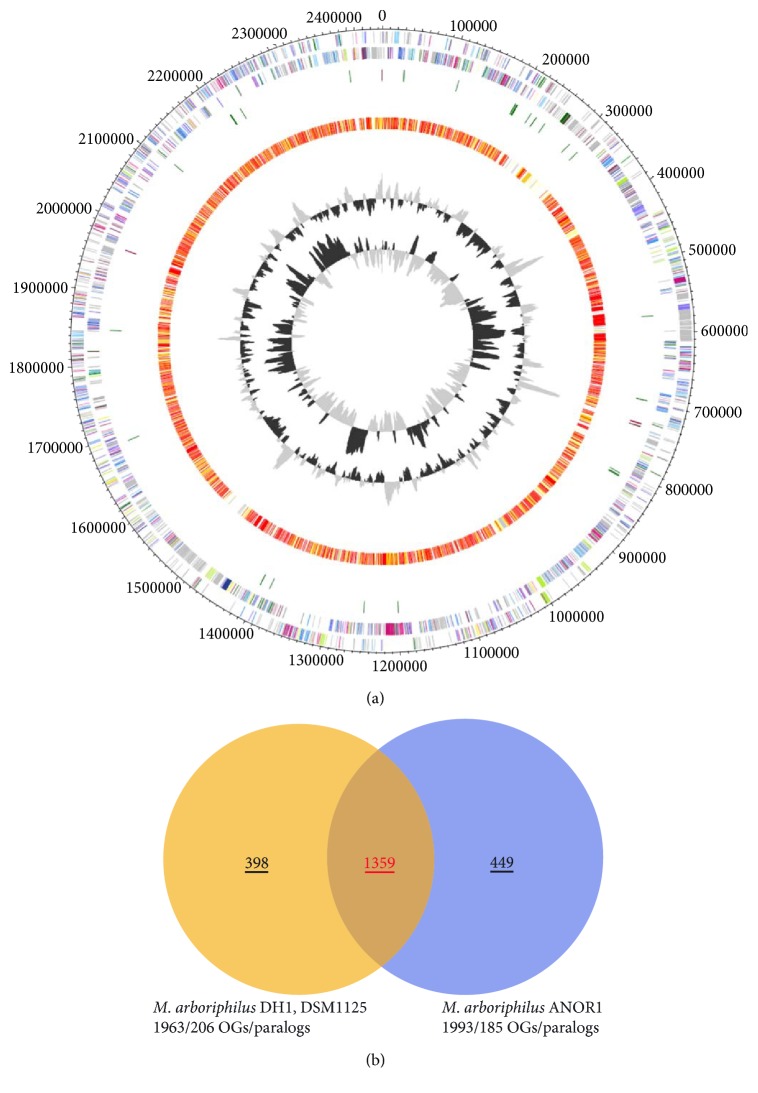
Genomic features of *Methanobrevibacter arboriphilus* strain DH1 and ANOR1. A circular representation of the *M. arboriphilus* strain DH1 and comparison with strain ANOR1 is shown in (a). The two outer rings represent both strands of the strain DH1 genome with genes coloured by COGs. The orange/red ring shows genes present in the genome of strain ANOR1 as determined by BLAST. The two inner rings represent GC content and GC skew of strain DH1. The numbers of genes shared by and specific for the two *Methanobrevibacter* strains are shown in (b). Contigs of the strain DH1 genome were aligned to the genome of strain ANOR1. Ortholog detection was done with the Proteinortho software version 4.26 [33] (BLASTp) with an identity cutoff of 50% and an *E* value of 1*e*^−10^. Visualization was done using Proteinortho results and DNAPlotter [52]. COG categories of the genes were extracted from IMG database entries of *M. arboriphilus* DH1. Colour code according to *E* values of the BLASTp analysis performed using Proteinortho 4.26. Grey, 1*e*^−20^ to 1; light yellow, 1*e*^−21^ to 1*e*^−50^; gold, 1*e*^−51^ to 1*e*^−90^; light orange, 1*e*^−91^ to 1*e*^−100^; orange, 1*e*^−101^ to 1*e*^−120^; red, >1*e*^−120^.

The *Methanobrevibacter arboriphilus* strain DH1 draft genome has been assembled into 40 contigs (>500 bp), with a N50 of 111,976 bp. Gene characteristics for plasmids were not detected in the assembled contigs. The completeness of the draft genome has been checked by using CheckM [28]. Here, a set of 188 ubiquitous and single copy marker genes was used for validation. Results revealed a 100% completeness of the genome, and in addition, it did not contain any contaminations. The GC content of the draft genome is 25.44%, which is almost identical to the GC content of the recently published draft genome of *Methanobrevibacter arboriphilus* strain ANOR1 (25.46%) [13], and is in accordance with the overall trend that most *Methanobrevibacter* and *Methanosphaera* species genomes tend to have low GC contents between ~24 and 33%, indicating that an early divergent evolution of these two genera from other Methanobacteriales groups may have occurred. Other general features of the genome and a comparison with properties of the *Methanobrevibacter arboriphilus* strain ANOR1 genome are shown in Table 1 and Figures 1(a) and 1(b).

The genome of strain DH1 also harbours a nonribosomal peptide synthetase (MBBAR_9c00060) and an adjacent phosphopantetheinyl transferase (MBBAR_9c00070). The nonribosomal peptide synthetase (NRPS) shares the highest sequence identity with a *Methanobacterium* sp. MB1 NRPS gene cluster (49%) and a *M. ruminantium* NRPS module (43%). NRPS modules are known to produce a wide variety of short-chain peptides, such as antibiotics or siderophores [34]. The function of these proteins in methanogens is currently not understood, and their regulation as well as the potential products of these modules in methanogens remains unclear.

### 3.2. Genes for Energy Metabolism and Methanogenesis

The gene repertoire required for the energy metabolism of *M. arboriphilus* strain DH1 is corresponding to that of the other autotrophic Methanobacteriales strain and has been described recently in detail for *Methanothermobacter marburgensis* [3, 35, 36]. *M. arboriphilus* strain DH1 is known to grow hydrogenotrophically, utilizing carbon dioxide and hydrogen [10]. The reduction of CO_2_ to methane proceeds via seven steps. Step one, the reduction of CO_2_ to formylmethanofuran, is catalysed by formylmethanofuran dehydrogenase (FwdABCDFGH). The genome encodes only the tungsten enzyme, while genes encoding the molybdenum-dependent isoenzyme appear to be absent. The following five steps in methanogenesis are catalysed by formylmethanofuran:H_4_MPT formyltransferase (Ftr), H_4_MPT^+^ cyclohydrolase (Mch), methylene-H_4_MPT dehydrogenase (Mtd), methylene-H_4_MPT reductase (Mer), and methyl-H_4_MPT:coenzyme M methyltransferase (MtrABCDEFGH). The final step is catalysed by methyl-coenzyme M reductase (McrABG). Potential genes encoding isoenzyme II of methyl-coenzyme M reductase (MrtABG) are absent from the draft genome. Similar observations have been made for other *Methanobrevibacter* species, for example, *Methanobrevibacter ruminantium*, and it has been speculated that the loss of the Mcr isoenzyme may represent an adaptation to growth at low levels of hydrogen [19, 37]. Gene IDs for genes encoding the enzymes involved in the seven methanogenesis reactions are given in Table S1 available online at https://doi.org/10.1155/2017/4097425. The majority of the deduced protein sequences share the highest sequence identity with those of the other Methanobacteriales.

The genome also harbours genes encoding a methanol:coenzyme M methyltransferase complex (MtaABC) and the activating protein (MapA), which indicates that the organism may potentially be capable of methylotrophic growth or could utilize methanol to some extent. The subunits of the MtaABC complex share high sequence identity (≥60%) with proteins from *Methanobacterium* sp. SWAN-1, *Methanobacterium lacus*, and *Methanobrevibacter smithii.* So far, no *Methanobrevibacter* species have been shown to grow on methanol alone or methanol and hydrogen as sole substrates for methanogenesis; however, in a recent study by Samuel et al., an increased expression of the mtaB gene in *M. smithii* was observed during cocolonization with *Bacteroides thetaiotaomicron* in mice [22].

### 3.3. Acetyl-CoA Synthesis

*M. arboriphilus* strain DH1 has been shown to grow autotrophically with CO_2_ and H_2_ in a mineral salts medium that contains vitamins [10]. Carbon assimilation in autotrophic methanogens occurs primarily through carbon monoxide dehydrogenase/acetyl-CoA decarbonylase complex. This complex (MBBAR_1c00380-MBBAR_00390) as well as some of its maturation factors is also encoded by the genome of strain DH1. This complex has thus far not been detected in the genomes of other rumen and intestinal *Methanobrevibacter* (with few exceptions, such as *Methanobrevibacter arboriphilus* sp. ANOR1 and some *Methanobrevibacter* species isolated from the termite hindgut [38]) and *Methanosphaera* species (*M. stadtmanae* and *M.* sp. WGK6 [16, 39]).

### 3.4. Nitrogen Metabolism

To the authors' knowledge, it has not been experimentally determined whether strain DH1 is capable of N_2_ fixation or if this strain is relying on transport systems to import ammonium or amino acids. Genome analysis revealed that both options are potentially available to this strain. The genome harbours genes encoding nitrogenase (MBBAR_1c00660-MBBAR_1c00730), most likely a molybdenum-iron type, which indicates that this organism may be capable of fixing atmospheric nitrogen. Nitrogenase has not been detected in the genomes of closely related intestinal *Methanobrevibacter* and *Methanosphaera* species (except for strain ANOR1 and some *Methanobrevibacter* species isolated from the termite hindgut). In addition to nitrogenase fixation, the genome also contains genes encoding ammonium transporters amt (MBBAR_5c00200, MBBAR_12c00350). Strain DH1 may also be able to import a number of different amino acids/oligopeptides via ABC-type transporters and may derive additional ammonium via deamination of amino acids. The latter would be in agreement with the observation that growth of some methanogens can be greatly improved by supplementation of the medium with amino acids [40].

### 3.5. Oxygen Detoxification Enzymes in *Methanobrevibacter arboriphilus* Strain DH1

**Figure 2:**
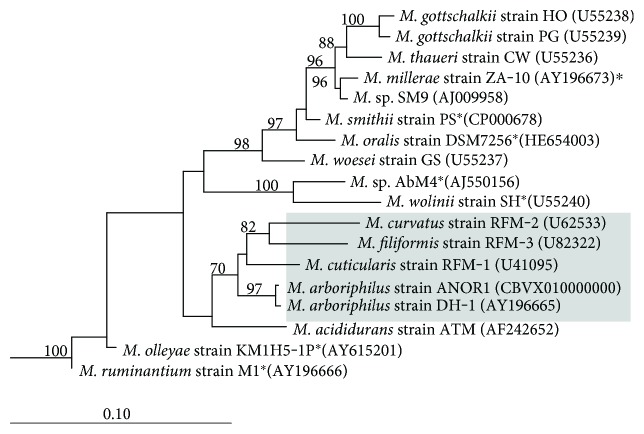
Phylogeny of *Methanobrevibacter* based on the 16S rRNA gene. Grey background indicates the presence of catalase (determined experimentally and/or by presence of the catalase gene). Asterisks behind species names indicate genomes where no apparent homologue of the *M. arboriphilus* catalase gene was detected by BLAST analysis. The tree was resampled 500 times, and only bootstrap values ≥70% are shown. The dendrogram was rooted with five Crenarchaeota sequences. The scale bar indicates 0.10 inferred nucleotide substitutions per position. *M.*: *Methanobrevibacter.*

The oxygen tolerance of *M. arboriphilus* has been attributed to several oxygen detoxification enzymes, which either protect from oxygen directly or its reactive species. The majority of these enzymes have also been detected in other Methanobacteriales species, but the finding of catalase activity in cell extracts of some *Methanobrevibacter* species was unexpected. The catalase gene has been cloned from *M. arboriphilus* strain AZ [15] but had not been known for strain DH1. Here, the catalase gene (MBBAR_24c00090) in strain DH1 was identified and its protein sequence revealed a 99% sequence identity to the enzyme of the AZ strain. The presence of catalase is restricted to only few of the *Methanobrevibacter* species, and the high protein sequence identity may indicate that the protein was acquired only fairly recently (Figure 2). Strain DH1, like other Methanobacteriales, is not capable of heme biosynthesis and requires therefore hemin supplementation to the medium to produce a functional catalase [15]. Analyses of the genome regions adjacent to the catalase gene did not reveal a transporter gene that could be involved in hemin uptake, and its identification awaits further functional characterization.

The strain DH1 genome harbours also genes encoding a number of other enzymes that are involved in the detoxification of oxygen, hydrogen peroxide, and superoxide. Some of these have been identified and characterized in closely related *Methanobrevibacter arboriphilus* strains, primarily strain AZ. Among these are the genes for rubrerythrin (MBBAR_3c00700), a predicted peroxidase [41], and the recently discovered and characterized F_420_H_2_ oxidase (MBBAR_3c00710) [42–44]. The proteins have 95% and 91% sequence identity, respectively, with corresponding proteins from strain AZ. The genome also encodes homologues of F_420_H_2_ oxidase (MBBAR_6c00420) and another rubrerythrin (MBBAR_6c00090). The latter appears to form a cotranscribed operon with a gene encoding a desulfoferredoxin (MBBAR_6c00080).

### 3.6. Genes for Adhesin-Like Proteins

One of the main objectives of this study was to determine the presence or absence of adhesin-like proteins. More than 50 gene-encoding ALPs were identified in the genome of *M. arboriphilus* strain DH1. This number of ALPs in the strain DH1 genome is higher than that in some host-associated *Methanobrevibacter* genomes. For comparison, *M. smithii* contains 48, *M.* sp. AbM4 29 (the currently known lowest number of ALPs in *Methanobrevibacter* genomes), and *M. ruminantium* 105 ALPs (the currently known highest number of ALPs in *Methanobrevibacter* genomes). This finding may indicate that at least some ALPs, if they function as adhesins, may have interaction partners other than host tissues. This hypothesis is corroborated by two findings: first, a recent analysis of a *M. ruminantium* ALP, Mru_1499, revealed that a domain of these adhesins may be important for the interaction between methanogens and protozoa and bacteria [8]. However, the large and diverse repertoire of methanogen ALPs encoded by some *Methanobrevibacter* genomes suggests that other interaction partners also need to be considered and that ALP subgroups/domains may have different host/target specificity. Second, smaller numbers of ALPs have also been encoded by genomes of other non-host-associated methanogens, for example, each 12 ALPs have been identified in the genomes of *Methanothermobacter marburgensis* and *M. thermautotrophicus* [2, 45, 46].

### 3.7. Differences between *Methanobrevibacter arboriphilus* Strains

The only currently available genome sequences of *M. arboriphilus* strains are those of strain DH1 (isolated from wetwood) and strain ANOR1. A comparative analysis of these strains' genomes was performed to identify features that are specific for either of the two strains and which may indicate specific adaptations to the environment they were isolated from. Overall, the genomes display a similar genome size and share a large number of genes (Figure 1). Among the shared genes are also some of those that would allow autotrophic growth, such as genes for CODH/ACS-complex and nitrogenase. Strain ANOR1 still awaits a physiological characterization which would help in determining if the enzymes are functional and whether the strain is capable of autotrophic growth.

The lack of genome information for other *M. arboriphilus* strains currently limits comparative analysis to only two strains. However, more evidence regarding differences between *M. arboriphilus* strains can be derived from previous physiological and biochemical studies. For example, it has been shown that cell extracts of strain AZ show activity of [Fe]-hydrogenase and the corresponding *hmd* gene has been cloned and heterologously produced in *Escherichia coli* [47]. There is no evidence for a *hmd* gene, *hmd* isoenzyme genes, or the recently described *hmd* co-occurring genes in strain DH1 [2]. Gene expression studies in other methanogens have shown that the expression of *hmd* and the isoenzymes may be regulated by hydrogen and/or also by nickel concentrations, for example, upregulation of *hmd* under low nickel concentrations [37, 48], but genetic studies have also revealed that hmd may not be required for growth on H_2_ and CO_2_ [49]. Depending on the environmental conditions some methanogens live in, these findings could help explain the presence and absence of *hmd* and co-occurring genes in genomes of some hydrogenotrophic methanogens. In the case of *M. arboriphilus*, these differences are even present at the strain level. At this stage, it is not possible to explain the reasons for these differences, but it is likely that these phenotypic differences represent specific adaptations to different environmental conditions. A pan-genome approach as recently undertaken for *M. smithii* [50] may shed light on the gene repertoire of these different strains and how they have adapted to different niches.

### 3.8. Is *M. arboriphilus* Strain DH1 Equipped to Survive in an Intestinal Environment?

Some of the genome features, for example, carbon monoxide dehydrogenase/acetyl-CoA decarbonylase complex and nitrogenase and the primary strain description and physiological characteristics, as well as its isolation source, support the finding that strain DH1 is a non-host-associated and autotrophic methanogen. However, it may need to be considered, whether strain DH1 may also be adapted to live in an intestinal environment as suggested by the presence of the high number of ALPs. Analyses of rumen and intestinal methanogens and their genomes have revealed some features, for example, sialic acid synthesis and bile acid hydrolases, which are associated with gut and/or rumen methanogens. The *Methanobrevibacter smithii* genome has been shown to encode genes predicted to be involved in sialic acid synthesis (neuA and neuB), but genes with only weak similarity to neuA and neuB (MMBAR_10c00110 and MBBAR_10c00100) were detected in strain DH1, and there is no experimental evidence that would support their function in sialic acid synthesis. Similar observations were made for bile acid hydrolases. Functional proof of bile acid hydrolases in *Methanosphaera stadtmanae* and *Methanobrevibacter smithii* has been provided, and the corresponding genes for the enzymes in the two genomes have been identified [51]; however, the *M. arboriphilus* draft genome does not harbour genes encoding closely related homologues of these enzymes. It needs to be taken into account that composition of bile acids and their metabolism may vary between hosts. Knowing the potential host may therefore be a prerequisite to determine the activity of such an enzyme in strain DH1.

It may also be necessary to consider intestinal environments other than the vertebrate intestinal tract as a habitat for strain DH1. The 16S rRNA gene of the strain shares high sequence identity with isolates from the termite hindgut (Figure 2), and also, some characteristic features, such as catalase, have been identified in *M. arboriphilus* and termite hindgut *Methanobrevibacter* strains [38]. Genome comparisons indicate that strain DH1 shares more genes with each of the *Methanobrevibacter* strains from the termite hindgut (1168–1293) than with species from the animal or human intestinal tract (1116–1151, excluding *M. arboriphilus* strain ANOR1). Some of the genes shared by strain DH1 and the termite hindgut *Methanobrevibacter* strains include genes encoding nitrogenase (present in all three termite hindgut *Methanobrevibacter* strains) and acetyl-CO-synthase/carbon monoxide dehydrogenase complex (present in *M. cuticularis* and *M. filiformis*) [38]. These findings suggest that it may be possible that *M. arboriphilus* strain DH1 could also be originating from an arthropod-associated microbiome. Alternatively, it could also indicate that *Methanobrevibacter* strains associated with the termite hindgut have conserved more genes for autotrophic growth in their genomes than the predominant *Methanobrevibacter* species from vertebrate intestinal tracts.

## 4. Conclusion

The genome of *M. arboriphilus* strain DH1 provides the first genome sequence of an autotrophic *Methanobrevibacter* species that is not associated with the gastrointestinal tract or rumen and serves as an important reference sequence for comparative genomics. The high abundance of adhesin-like proteins in this *Methanobrevibacter* genome was unexpected and raises further questions about their function and interaction partners. These results reveal that ALPs may be a common feature of some methanogen clades, but they may not be involved in the interaction between the methanogen and the surface of host tissues to the extent as previously assumed.

## Supplementary Material

Table S1. ANIm and aligned percentage. Table S2: DDH-analysis of *Methanobrevibacter arboriphilus* strains. Table S3. Genes encoding proteins involved in methanogenesis.

## Figures and Tables

**Table 1:** 

DNA, total number of bases	2,445,031	100.00%
DNA coding number of bases	1,825,159	74.65%
DNA G + C number of bases	622,051	25.44%^1^
DNA scaffolds	40	100.00%
CRISPR count	9	
Gene total number	2007	100.00%
Protein-coding genes	1959	97.61%
RNA genes	48	2.39%
rRNA genes	7	0.35%
5S rRNA	3	0.15%
16S rRNA	2	0.10%
23S rRNA	2	0.10%
tRNA genes	38	1.89%

^1^GC percentage shown as count of G's and C's divided by the total number of bases. The total number of bases is not necessarily synonymous with a total number of G's, C's, A's, and T's.
