# Supplementary material for: The Draft Genome of the Non-Host-Associated Methanobrevibacter arboriphilus Strain DH1 Encodes a Large Repertoire of Adhesin-Like Proteins
Source: Archaea. 2017 May 28;2017:4097425. doi: 10.1155/2017/4097425 (PMC5467289; doi:10.1155/2017/4097425)
Supplement: Supplementary file 1 — Table S1. ANIm and aligned percentage. Table S2: DDH-analysis of Methanobrevibacter arboriphilus strains. Table S3. Genes encoding proteins involved in methanogenesis. [file 4097425.f1.pdf]

Table S1. ANIm and aligned percentage

	MMBAR.fas	MBB_JCM13429.fna	JCM_03232017.fna	ANOR1.assembled.fna
MMBAR.fas	*	99.59 [93.68]	99.59 [93.68]	93.25 [78.71]
MBB_JCM13429.fna	99.59 [99.89]	*	100.00 [100.00]	93.39 [80.15]
JCM_03232017.fna	99.59 [99.89]	100.00 [100.00]	*	93.39 [80.15]
ANOR1.assembled.fna	93.26 [85.26]	93.40 [81.20]	93.40 [81.20]	*

Table S2: DDH-analysis of *Methanobrevibacter arboriphilus* strains

Query genome	Reference genome	Formula 1<br>DDH	Model C.I.	Bootstrap C.I.	Distance	Prob. DDH >= 70%
M. arboriphilus DH1, DSM 1125	M. arboriphilus ANOR1	64.10	[61 - 67.1%]	63.7 - 64.6%	0.3942	58.38
M. arboriphilus DH1, DSM 1125	M. arboriphilus DH1, JCM 13429	74.90	[71.7 - 77.9%]	72.8 - 77%	0.3088	85.36

Table S3. Genes encoding proteins involved in methanogenesis

Locus_tag	Protein name	EC Number	gene name
MBBAR_3c01660	formylmethanofuran dehydrogenase, subunit A	EC 1.2.99.5	fwdA
MBBAR_3c01650	formylmethanofuran dehydrogenase, subunit B	EC 1.2.99.5	fwdB
MBBAR_3c01670	formylmethanofuran dehydrogenase, subunit C	EC 1.2.99.5	fwdC
MBBAR_3c01640	formylmethanofuran dehydrogenase, subunit D	EC 1.2.99.5	fwdD
MBBAR_3c01620	formylmethanofuran dehydrogenase, subunit F	EC 1.2.99.5	fwdF
MBBAR_3c01630	formylmethanofuran dehydrogenase, subunit G	EC 1.2.99.5	fwdG
MBBAR_3c01610	formylmethanofuran dehydrogenase, subunit H	EC 1.2.99.5	fwdH
MBBAR_1c01730	formylmethanofuran-tetrahydromethanopterin formyltransferase	EC 2.3.1.101	ftr
MBBAR_6c00310	methenyltetrahydromethanopterin cyclohydrolase	EC 3.5.4.27	mch
MBBAR_12c00400	methylenetetrahydromethanopterin dehydrogenase	EC 1.5.99.9	mtd
MBBAR_1c00020	5,10-methylenetetrahydromethanopterin reductase	EC 1.5.98.2	mer
MBBAR_2c00380	tetrahydromethanopterin S-methyltransferase, subunit A	EC 2.1.1.86	mtrA
MBBAR_2c00390	tetrahydromethanopterin S-methyltransferase, subunit B	EC 2.1.1.86	mtrB
MBBAR_2c00400	tetrahydromethanopterin S-methyltransferase, subunit C	EC 2.1.1.86	mtrC
MBBAR_2c00410	tetrahydromethanopterin S-methyltransferase, subunit D	EC 2.1.1.86	mtrD
MBBAR_2c00420	tetrahydromethanopterin S-methyltransferase, subunit E	EC 2.1.1.86	mtrE
MBBAR_2c00370	tetrahydromethanopterin S-methyltransferase, subunit F	EC 2.1.1.86	mtrF
MBBAR_2c00360	tetrahydromethanopterin S-methyltransferase, subunit G	EC 2.1.1.86	mtrG
MBBAR_2c00350	tetrahydromethanopterin S-methyltransferase, subunit H	EC 2.1.1.86	mtrH
MBBAR_2c00430	methyl-coenzyme M reductase, subunit alpha	EC 2.8.4.1	mcrA
MBBAR_2c00470	methyl-coenzyme M reductase, subunit beta	EC 2.8.4.1	mcrB
MBBAR_2c00440	methyl-coenzyme M reductase, subunit gamma	EC 2.8.4.1	mcrG
MBBAR_6c00020	methanol:coenzyme M methyltransferase		mtaA
MBBAR_6c00050	methanol:coenzyme M methyltransferase		mtaB
MBBAR_6c00040	methanol:coenzyme M methyltransferase		mtaC
MBBAR_6c00030	methyltransferase activation protein, large subunit		mapA
